# Helminth parasites of Galápagos mammals: a new cestode of the genus *Raillietina* from the endemic rice rat *Nesoryzomys swarthi* and a summary of parasites from both endemic and invasive rodents

**DOI:** 10.1017/S0031182025000083

**Published:** 2025-02

**Authors:** Scott L. Gardner, Emma K. Chesley, Michael C. Friedle, Altangerel T. Dursahinhan

**Affiliations:** H.W. Manter Laboratory of Parasitology, University of Nebraska State Museum, University of Nebraska-Lincoln, Lincoln, NE, USA

**Keywords:** Cestoda, Darwin, Galápagos, *Hymenolepis*, *Nesoryzomys swarthi*, *Protospirura*, *Raillietina*, rodents, Symbiotype, *Taenia* spp

## Abstract

In this first report of endoparasites from endemic land-mammals of the Galápagos Islands, we describe a new species of cestode of the genus *Raillietina* (Cyclophyllidea: Davaineidae) from a species of *Nesoryzomys* and summarize the extent of helminth parasitism in both oryzomyine endemics and introduced species of *Rattus*. Up to the current time, no helminth parasites have been reported from rodents of the Galápagos, and little work has yet been done describing and synthesizing Galápagos parasite diversity. In historical times, several species of autochthonous rodents have occupied the islands including: *Nesoryzomys narboroughi* Heller 1904, *N. fernandinae* Hutterer and Hirsch 1979, *N. swarthi* Orr, 1938, and *Aegialomys galapagoensis* (Waterhouse, 1839). Colonization of the islands by humans brought 3 known species of synanthropic rodents: *Rattus rattus, R. norvegicus*, and *Mus musculus* which are suspected to have caused the extinction of at least 3 other oryzomyines in historical times.

## Graphical abstract

**Figure:**
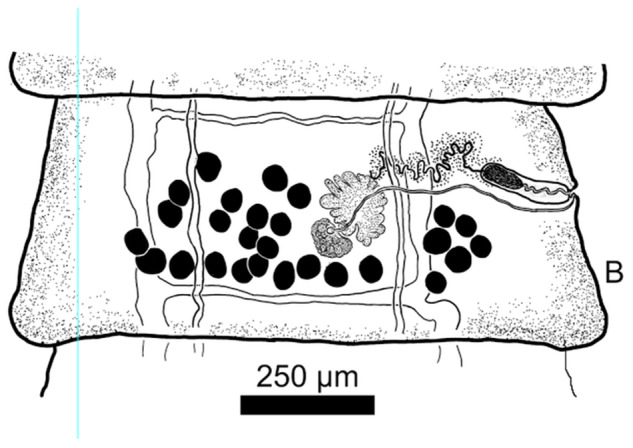

## Introduction

The organisms living in and around the Galápagos archipelago are some of the most well-studied life-forms on our planet relative to development and understanding of natural selection as the ultimate driver of organismal evolutionary change and speciation (Lack, 1940; Boag and Grant, 1981; Schluter and Grant, 1984; Gould, 2002; Lamichhaney *et al*., 2018; Zink, 2002). Interestingly, Darwin (1845) wrote only a few pages about his time in the Galapagos where he summarized his scientific collecting work while there and where he superficially noted the geological, climatic, zoological and botanical diversity of the islands. It was only later, after he had returned to England and distributed his collection of biological specimens accumulated during his voyage on the HMS Beagle to various experts at museums that his colleagues in those museums soon let him know that every island of the Galapagos archipelago was inhabited by different species of animals and plants. This finding both stunned and dismayed Darwin because when he was initially collecting specimens on the various islands, he had mixed together his scientific collections from at least 2 of the islands. He stated (Darwin, 1845, pp. 393–394):

*I have not yet noticed by far the most remarkable feature in the natural history of this archipelago; it is, that the different islands to a considerable extent are inhabited by a different set of beings. My attention was first called to this fact by the vice governor, Mr. Lawson, declaring that the tortoises differed from the different islands and that he could with certainty tell from which island any one was brought. I did not for some time pay attention to this statement and I had already partly mingled together the collections from two of the islands. I never dreamed that islands, about fifty or sixty miles apart, and most of them in sight of each other, formed of precisely the same rocks, placed under a quite similar climate, rising to a nearly equal height, would have been differently tenanted; but we shall soon see that this is the case.*

The Galápagos Islands, rising from the floor of the Pacific Ocean on the equator, are volcanic in origin, being formed from the action of a stationary sub-crustal magmatic plume or hot spot situated under an easterly moving piece of the earth’s crust called the Nazca plate (Holden and Dietz, 1972; Geist et al., 2014a, b). In this archipelago, the current estimate of the maximum age of the easternmost islands is around 3.5 million years (White *et al.,* 1993; Christie et al., 1992) with an estimated minimum age of around 500,000 years for the islands to the west (Christie et al., 1992; Harpp and Geist, 2018). The islands are biologically isolated being located on the equator about 950 km west of continental South America. The expedition of biological exploration led by Darwin commenced in the Galápagos on September 15, 1835, while the collecting expeditions (directed and led by Dr Robert C. Dowler, Angelo State University, San Angelo, Texas) that ultimately led to the discovery of the parasites identified and described herein occurred in 1999. Dowler et al. (2000) reported on the collecting trips to the Galápagos that occurred in 1995 and 1997 where endemic rodents, that had been considered extinct, were rediscovered. The collecting trip in 1999 was informed by the previous 2 expeditions and specimens of both parasites and their mammalian hosts were preserved as museum specimens (Dowler et al., 2000).

Knowledge of the approximate ages of individual islands and thus the history of the geological evolution of the emergence of the Galápagos chain into dry-land habitats has a direct impact on our ability to understand the potentially reciprocal biological evolution of the flora and fauna of the islands. As such, one of the best-known examples of natural selection in action comes from studies of the Galápagos finches, which are members of the Tanager family Thraupidae Cabanis, 1847, and the evolution of the 13 species of Darwin’s finches appears to have been from an initial colonization event that occurred around 2–3 million years ago (Sato et al., 1999; Abzhanov, 2010). Interestingly, the divergence time among some of the 7 species of the endemic Galápagos lava lizards of the genus *Microlophus* Duméril and Bibron, 1837, has been estimated to be as old as 9 million years (Rassmann, 1997). Since this estimated species divergence time for the species of lizards in the Galápagos is older than the oldest known island, various hypotheses relative to the ages of the islands and arrival times into the Galápagos of animal groups have been proposed, but the dynamic nature of appearance and disappearance of these volcanic islands plays a large part in forming this complex biota (Heads, 2014).

If emergent volcanoes existed over the Galápagos magmatic hot spot prior to the emergence and establishment of the current islands, then much of the Galápagos biota could have evolved on these past islands and then transferred or hopped to the new islands when they arose above the surface of the sea, thus explaining species divergence times older than the current islands themselves (Rassmann, 1997; Heads, 2014). The presence of sub-surface seamounts situated southeast of the islands suggests there have been islands forming over the Galápagos magmatic hot spot for at least 14 million years and these now submerged islands may have served as stepping stones or initial landing spots for sweepstakes dispersalists from mainland habitats (Christie et al., 1992; Hoernle et al., 2002). Initial sweepstakes dispersal via oceanic rafting from the mainland is likely how the Galápagos archipelago first saw the arrival of rice rats, as they are hypothesized to be good dispersers across saltwater (Castañeda-Rico et al., 2019).

Despite the importance of the endemic fauna and flora of the Galápagos archipelago to the development of the theory of speciation and subsequently the theoretical aspects of evolution (Darwin, 1859; Lack, 1940, 1947; Grant and Grant, 1992; Grant, 1986), relatively little work has been done on the evolutionary biology and phylogeny of parasites of vertebrates of these islands. Most published studies related to parasite diversity there are biased towards the avifauna and their ectoparasites with a significant blank in the research literature regarding helminth diversity of avian hosts, as shown in Table 1. Up to the current time, we are not aware of any publications discussing, describing, or even mentioning the diversity of helminth parasites from either the autochthonous land mammals or from any of the introduced synanthropic rodents which currently include only species of *Rattus* and *Mus*. Even so, some work on the parasites of vertebrates has been accomplished and Bataille et al. (2018) published a summary of all known ecto- and endoparasites of vertebrates of the Galápagos biota and provided a discussion of their likely mode of arrival in the island chain. Their review shows that species of the phylum Apicomplexa make up the majority of the documented endoparasites of the Galápagos endemic avifauna (Table 1). Gettinger et al. (2011) published on mites of the family Laelapidae and described a new species from *Aegialomys galapagoensis* (Waterhouse, 1839). From birds, Jiménez-Uzcátegui et al. (2015).reported several helminths from the Waved Albatross, *Phoebastria irrorata* (Salvin, 1883) collected from the island of Española including unidentified species of Nemata (genus *Contracaecum* Railliet and Henry, 1912), a species of cestode assignable to *Tetrabothrius* Rudolphi, 1819, and a species of trematode in the genus *Cardiocephaloides* Sudarikov, 1959. From the islands of Isabela and Fernandina, an unidentified species of *Contracaecum* and an unidentified trematode of the family Heterophyidae were reported from the flightless cormorant, *Nannopterum harrisi* (Rothschild, 1898) (see Carrera-Jativa et al., 2014) and species of *Contracaecum* and the trematode *Renicola* sp. were reported from the Galápagos brown pelican, *Pelecanus occidentalis* Linnaeus, 1766, from several islands (Table 1) (Parker et al., 2006). Finally, an unidentified trematode was reported from the Galápagos rail, *Laterallus spilonota* (Gould, 1841) (see Bataille et al., 2018). All of these helminth parasites are known to have narrow host-ranges (*sensu* Agosta, 2006, 2022) occurring only in birds and as will be seen herein, there have been no documented cases of ecological fitting (Janzen, 1980; Agosta, 2006) involving birds and rodents now occurring on the islands.

**Table 1.** Recorded endoparasites found present in Galápagos endemic vertebrates, including birds, mammals and reptiles

	Locality	Parasite	Reference
**Mammals**			
*Nesoryzomys swarthi*	Santiago	*Raillietina dowleri* n. sp.	This paper
*Aegialomys galapagoensis*	Santa Fe	*Physaloptera calnuensis*	This paper
		*Mastophorus muris*	
*Nesoryzomys fernandinae*	Fernandina	*Mastophorus muris*	This paper
*Nesoryzomys narboroughi*	Fernandina	*Raillietina*	This paper
*Rattus rattus*	Santiago	*Hymenolepis diminuta*	This paper
	Santa Cruz	*Mastophorus muris*	This paper
	San Cristobal	*Taenia taeniaeformis*	This paper
		*Mastophorus muris*	
		*Hymenolepis diminuta*	
		*Protospirura numidica*	
	Isabela	*Physaloptera calnuensis*	This paper
		*Taenia taeniaeformis*	
		*Hymenolepis diminuta*	
*Zalophus wollebaeki*	Santa Cruz	*Philophthalmus zalophi*	Dailey et al., 2005
	San Cristóbal	*Philophthalmus zalophi*	Walden et al., 2018
		Lungworms	
		Nematodes	
		Cestodes	
**Birds**			
*Geospiza* spp.	Santa Cruz	*Isopora geospizae*	McQuistion and Wilson, 1989
*Spheniscus mendiculus*	Isabela	*Plasmodium, Haemoproteus*	Levin et al., 2009
	Marielas		
	Fernandina		
	Bartolome		
	Santiago		
	Floreana		
	Santa Cruz		
	Fernandina	*Chlamydophila psittaci*	Parker et al., 2006
	Floreana		
	Isabela		
	—	*Toxoplasma gondii*	Bataille et al., 2018
*Phoebastria irrorata*	Española	*Contracaecum*,	Jiménez-Uzcátegui et al., 2015
		*Tetrabothrius*,	
		*Cardiocephaloides*	
*Zenaida galapagoensis*	Santiago	*Haemoproteus*,	Santiago-Alarcon et al., 2010; Parker et al., 2006
		*Chlamydophila psittaci*	
	Santa Cruz		
	Santa Fe		
	Española		
	San Cristóbal		
	Genovesa		
	Darwin		
	Wolf		
	Fernandina	*Chlamydophila psittaci*	Parker et al., 2006
	Floreana		
	Isabela		
	Marchena		
	Pinta		
	Pinta		
	—	*Eimeria palumbi*	Bataille et al., 2018
*Nannopterum harrisi*	Isabela	*Contracaecum*,	Carrera-Jativa et al., 2014
		Heterophyidae	
	Fernandina		
	Fernandina	*Chlamydophila psittaci*	Parker et al., 2006
	Isabela		
	—	*Toxoplasma gondii*	Bataille et al., 2018
*Buteo galapagoensis*	Española	*Trypanosoma* sp.	Parker et al., 2006
	Fernandina		
	Isabela		
	Marchena		
	Pinta		
	Santa Fe		
	Santiago		
*Pelecanus occidentalis*	Española	*Contracecum, Renicola*	Parker et al., 2006
	Fernandina		
	Floreana		
	Isabela		
	Santa Cruz		
	Santiago		
*Mimus parvulus*	—	*Polysporella genovesae*	Bataille et al., 2018
*Laterallus spilonota*	—	Trematode	Bataille et al., 2018
*Creagrus furcatus*	Española	*Haemoproteus* sp.	Parker et al., 2006
	Fernandina		
	Floreana		
	Genovesa		
	Isabela		
	Marchena		
	Pinta		
	San Cristobal		
	Santa Cruz		
	Santa Fe		
	Santiago		
*Fregata magnificens*	Genovesa	Undermined *Haemoproteus* sp.	Parker et al., 2006
	Isabela		
	San Cristobal		
**Reptiles**			
*Chelonoidis niger*	Santa Cruz	Nematodes	Fournié et al., 2015
	Isabela		
	San Cristóbal		
*Chelonoidis niger*	Santa Cruz	*Eimeria*	Couch et al., 1996
*Microlophus spp.*	Española	*Eimeria, Isopora*	Couch et al., 1996
	Santa Cruz		
	Santa Fe		
	San Cristóbal		
	Seymour Norte		
	Santa Cruz	*Schellackia* or *Sarcocystis*	Ayala and Hutchings, 1974
*Amblyrhynchus cristatus*	Fernandina	*Hepatozoon*	Bataille et al., 2012
	Isabela		
*Conolophus subcristatus*	Wolf	*Hepatozoon*	Onorati et al., 2017
		Nematodes	Cuckler AC, 1938

Curiously, up to the current time, the only reported endoparasite from Darwin’s finches is the coccidian *Isospora geospizae* (McQuistion and Wilson, 1989), although there was also an anecdotal account by Grant who said: ‘*… virtually nothing is known about parasites and disease beyond the discovery of parasitic worms in a cactus finch (Salvin 1877), the occasional observation of worms in the feces of ground finches (D. Schluter, pers. comm.),* …’ (Grant, 1986, p. 65).

Similar to the avifauna of the area, Galápagos reptiles have also been found infected with species of Apicomplexa (Couch et al., 1996), and it is interesting that a significant literature has developed around the diversity of pinworms (Nemata: Oxyurida) and other nematodes of the Galápagos tortoise species group, see Petter (1966); Petter and Douglas (1976); Bouamer and Morand (2006), Walton AC (1942), and Fournié et al. (2015) and references therein.

McIntosh (1939) described *Infidum luckeri* McIntosh, 1939, a digenetic trematode recovered from the gall bladder of a specimen of the Jubo snake, *Phylodryas hoodensis* (Van Denburgh, 1912) that died in the US National Zoo (snake specimen No. 7485, parasite specimen – former United States National Parasite Collection (USNPC) Helm. Coll. No. 43409) and was collected most likely either from the island of Española or from Gardner Island, near the island of Española by members of the 1938 Presidential Cruise; these are the only 2 islands from which this species of snake is known (Thomas, 1997).

### Parasites of mammals

To our knowledge, the Galapagos sea lion, *Zalophus wollebaeki* Sivertsen, 1953, is the only mammal in the archipelago reported to be infected with endoparasites prior to the current study; here, individual sea lions were reported to host the eye fluke, *Philophthalmus zalophi* (Dailey et al., 2005) (Digenea: Philophthalmidae) collected from the islands of Santa Cruz and San Cristobál. In addition, from these sea lions, ascaridoid nematode eggs, other unidentified juvenile nematodes, coccidian oocysts and some cestode eggs, identified as belonging to the order Pseudophyllidea were reported (Dailey et al., 2005; Walden et al., 2018). Mites and lice recovered while examining sea lions for the trematode study are also deposited in the former USNPC, but no identifications were attempted (Dailey et al., 2005).

Endemic rodents known from the Galápagos Archipelago include species in the genera *Nesoryzomys* Heller 1904, *Aegialomys* Weksler *et al*., 2006, and *Megaoryzomys* (Lenglet and Coppois, 1979). Species of *Nesoryzomys* and *Aegialomys* are placed in the tribe Oryzomyini (Rodentia: Cricetidae) (see Lenglet and Coppois, 1979; Salazar-Bravo *et al*., 2016; Ronez et al., 2021). During historical times, 6 species of endemic rodents are known to either have occurred on or currently inhabit various islands of the Galápagos (Tables 2 and 3). Two species of endemic Galápagos rice rats, including *Nesoryzomys indefessus* (Thomas, 1899) and *N. darwini* Osgood, 1929, have recently (IUCN, 2019) been declared extinct by the International Union for the Conservation of Nature. Presumably viable populations of 4 other species are still extant, but all are under extreme pressure of anthropogenically mediated imminent obliteration. These species include: *Nesoryzomys narboroughi* Heller, 1904, *N. fernandinae* Hutterer and Hirsch, 1979, *N. swarthi* Orr, 1938, and *Aegialomys galapagoensis* (Waterhouse, 1839) (see also Prado and Percequillo (2018) and [Tables 2 and 3]). Interestingly, Percequillo et al. (2021) show that a divergence time between the taxa that gave rise to the genera *Nesoryzomys* and *Aegialomys* was during Pleistocene time and based on this multi-locus phylogenetic analysis, it appears that precursors of the species of these 2 genera entered into the Galapagos simultaneously and did not evolve from a common ancestor in the islands.

**Table 2.** Status of all known Galápagos rodents recorded in the literature

Species	Status	Reference
*Rattus rattus* (Linnaeus, 1758)	Invasive	Harper and Carrion, 2011
*Rattus norvegicus* (Berkenhout, 1769)	Invasive	Harper and Carrion, 2011
*Mus musculus* Linnaeus, 1758	Invasive	Harper and Carrion, 2011
*Nesoryzomys narboroughi* Heller, 1904	Endemic extant	Castañeda-Rico et al., 2019
*Nesoryzomys fernandinae* Hutterer & Hirsch, 1979	Endemic extant	Castañeda-Rico et al., 2019
*Nesoryzomys swarthi* Orr, 1938	Endemic extant	Castañeda-Rico et al., 2019
*Aegialomys galapagoensis* (Waterhouse, 1839)	Endemic extant	Castañeda-Rico et al., 2019
*Nesoryzomys indefessus* (Thomas, 1899)	Endemic extinct	Castañeda-Rico et al., 2019
*Nesoryzomys darwini* Osgood, 1929	Endemic extinct	Castañeda-Rico et al., 2019
Megaoryzomys curioi* (Niethammer, 1964)	Endemic extinct	Ronez et al., 2021

* *Megaoryzomys curioi* has only been identified from remnant skeletal material (Ronez et al., 2021).

**Table 3.** Distribution and status of rodents recorded on each island of the Galápagos archipelago

Island	Rodent	Status	Reference
Isabela	*Rattus rattus*	Extant	Patton et al., 1975
	*Mus musculus*	Extant	Key and Muñoz Heredia, 1994
	*Rattus norvegicus*	Extant	Harper and Carrion, 2011
	*Nesoryzomys spp.*	Extinct	Steadman and Ray, 1982
	*Megaoryzomys curioi*	Extinct	Key and Muñoz Heredia, 1994
Santa Cruz	*Rattus rattus*	Extant	Dowler et al., 2000
	*Rattus norvegicus*	Extant	Key and Muñoz Heredia, 1994
	*Mus musculus*	Extant	Key and Muñoz Heredia, 1994
	*Nesoryzomys indefessus*	Extinct	Castañeda-Rico et al., 2019
	*Nesoryzomys darwini*	Extinct	Castañeda-Rico et al., 2019
	*Megaoryzomys curioi*	Extinct	Ronez et al., 2021
San Cristóbal	*Rattus rattus*	Extant	Key and Muñoz Heredia, 1994
	*Rattus norvegicus*	Extant	Key and Muñoz Heredia, 1994
	*Mus musculus*	Extant	Key and Muñoz Heredia, 1994
	*Aegialomys galapagoensis*	Extinct	Castañeda-Rico et al., 2019
Fernandina	*Nesoryzomys fernandinae*	Extant	Castañeda-Rico et al., 2019
	*Nesoryzomys narboroughi*	Extant	Castañeda-Rico et al., 2019
Santiago (San Salvador)	*Rattus rattus*	Extant	Dowler et al., 2000
	*Mus musculus*	Extant	Dowler et al., 2000
	*Nesoryzomys swarthi*	Extant	Castañeda-Rico et al., 2019
Floreana	*Rattus rattus*	Extant	Patton et al., 1975
	*Mus musculus*	Extant	Key and Muñoz Heredia, 1994
Santa Fe	*Aegialomys galapagoensis*	Extant	Castañeda-Rico et al., 2019
Baltra (South Seymour)	*Rattus rattus*	Eradicated	Harper and Carrion, 2011
	*Mus musculus*	Extant	Harper and Carrion, 2011
	*Nesoryzomys indefessus*	Extinct	Castañeda-Rico et al., 2019
Seymour Norte	*Rattus rattus*	Eradicated	Harper and Carrion, 2011
	*Mus musculus*	Eradicated	Harper and Carrion, 2011
Pinzón	*Rattus* rattus	Extant	Key and Muñoz Heredia, 1994
Rábida	*Rattus norvegicus*	Eradicated	Key and Muñoz Heredia, 1994

Invasive rodents that have successfully colonized various islands in the Galapagos include the black rat, *Rattus rattus* (Linnaeus, 1758), Norway rat, *Rattus norvegicus* (Berkenhout, 1769) and house mouse, *Mus musculus* Linnaeus, 1758. All 3 species arrived on the islands by accompanying humans, with *R. rattus* founding successful invading populations at least 3 times, with the first occurring between the 17th and 18th centuries (Harper and Carrion, 2011; Phillips et al., 2012) (see Table 3).

The current report provides information derived from a survey where both endemic and invasive rodents in the Galápagos were collected and preserved as museum specimens while giving a description and comparisons of a new species of cestode of the genus *Raillietina*.

This is the first report of species of *Raillietina* Fuhrman, 1920, (Cyclophyllidea: Davaineidae) from endemic rodents in the Galápagos. The only other species of *Raillietina* reported from vertebrates on the islands is *R. echinobothrida* Mégnin, 1880, from domestic chickens on both San Cristobal and Santa Cruz islands (Gottdenker et al., 2005); this species is known to use both beetles and ants as intermediate hosts (Panich et al., 2021) and is not known from any endemic Galápagos vertebrates. Interestingly, 2 species of ants of the genus *Pheidole* Westwood, 1839, were demonstrated to be the intermediate host for *R. loeweni* (Cestoda) from the black-tailed jackrabbit, *Lepus californicus* Gray, 1837, in Kansas (Bartel, 1965) and at least 1 species of this genus of ant appears endemic to the Galápagos (Herrera et al., 2024).

## Materials and methods

All rodents were captured using Sherman^TM^ and Tomahawk^TM^ live traps baited with a mixture of dried rolled oats and peanuts. After capture, specimens were euthanized using chloroform, examined for arthropod (ecto-) and helminth (endo-) parasites, prepared as museum specimens, and transported back to museums in the USA. The pleural and peritoneal cavities were opened and examined for gross evidence of parasites and the intestines were removed, opened and the contents were searched for parasites. All parasites found were fixed in 10% formalin, transported, and stored in a solution of 10% formalin until study. At time of study, specimens of nematodes and cestodes were in placed in 70% ethanol and stored in this solution until staining or clearing. For morphological examination of nematodes, all specimens were transferred to 70% ethanol, rinsed several times in fresh 70% ethanol, cleared for 24 h in lactophenol and mounted in lactophenol on a standard microscope slide under a no. 1 coverslip with a small piece of museum-quality tag-paper under 1 edge of the cover slip to keep the cover slip from squashing the specimen over time. Specimens so prepared were then studied with a Zeiss Axiophot^TM^ digital microscope. All cestodes preserved in the field and transferred to the Manter Laboratory were rinsed several times in 70% ethanol times, stained in Semichon’s Acetic Carmine, destained in 70% acid alcohol, neutralized in 70% ethanol with a few drops of ammonium hydroxide, dehydrated to 100% ethanol in a series of ethanol baths ranging from 70%–85%–95%–100% ethanol (with 2 rinses in 100% with an interval of 20 min), cleared in terpineol, rinsed quickly in xylene and mounted on a microscope slide under a No. 1 cover slip in gum Damar. Larval cestodes found in the livers of *Rattus* spp. were stained in Semichon’s Acetic Carmine and cleared in lactophenol. To study the hooks of the larval Taeniids, the rostellum was removed and hooks were spread in lactophenol with pressure of a pencil eraser under a 15 mm square coverslip on a standard microscope slide. For the new species of cestodes reported herein, holotype and paratype specimens were deposited in the Parasite Collection of the Harold W. Manter Laboratory of Parasitology, the University of Nebraska-Lincoln (HWML). All helminths recovered and studied are also deposited in the HWML Parasite collections. HWML numbers are given in results.

## Results

Endemic species studied in this paper included individuals of *Nesoryzomys swarthi* obtained from near La Bomba, Santiago Island (0°11.21ʹS; 90°42.04ʹW) while individuals of both *Nesoryzomys narboroughi* and *N. fernandinae* were collected at Cabo Douglas on Fernandina Island (1°18.24ʹS; 91°39.14ʹW). Specimens of *Aegialomys galapagoensis* were obtained from suitable habitats on Santa Fe Island (0°48.21ʹS; 90°2.45ʹW). Invasive species studied included *Rattus rattus* collected on Volcan Wolf (0°3.96ʹN; 91°24.18ʹW) and Cerro Azul (0°55ʹ42.0954ʹʹN; 91°23ʹ36.9ʹʹW) of Isabela Island while specimens of *Rattus norvegicus* were collected on Rábida Island (0°24ʹ17.3874ʹʹ;90°42ʹ28.0ʹʹ).

Twelve individuals of each species of endemic rodents were collected and processed as museum specimens. Additionally, 22 individuals of *R. rattus* and 7 individuals of *R. norvegicus* were collected, processed, and examined for ecto- and endoparasites. See Table 4 for data on prevalence and numbers for individual species of nematode and cestode parasites recovered. Tapeworms identified as *Hymenolepis diminuta* Rudolphi, 1819, (Cestoda: Hymenolepididae) were found in both *R. rattus* and *R. norvegicus.* No tapeworms of the genus *Hymenolepis* were found in endemic rodents.

**Table 4.** Prevalence of endoparasites in rodent species collected by Dr Robert Dowler in 1999

Species	Total examined	No. infected	Percentage infected	Parasites found	Animals infected	Percentage of individuals infected	Percentage of total individuals
*Nesoryzomys swarthi*	12	5	41·6	*Raillietina*	5	41·6	41·6
*Nesoryzomys fernandinae*	12	6	50	*Mastophorus muris*	6	50	50
*Aegialomys galapagoensis*	12	2	16·7	*Physaloptera calnuensis*	1	50	8·33
				*Mastophorus muris*	1	50	8·33
*Nesoryzomys narboroughi*	12	0	0	—	—	—	—
*Rattus norvegicus*	7	0	0	—	—	—	—
*Rattus rattus*	22	22	100	*Hymenolepis diminuta*	5	22·73	22·73
				*Physaloptera calnuensis*	1	4·55	4·55
				*Taenia taeniaeformis*	8	36·36	36·36
				*Mastophorus muris*	4	18·18	18·18
				*Protospirura numidica*	1	4·55	4·55

During this work, a new species of the cestode genus *Raillietina*, was found to occur in the small intestines of 5 specimens of *N. swarthi* collected at La Bomba, on Isla Santiago. Importantly, none of the *Rattus* that were examined were found to harbour specimens of *Raillietina*, although, as noted, these rodents did harbour the almost ubiquitous *Hymenolepis diminuta.*

Following is the description of a new species of *Raillietina*. Measurements are given in micrometres (µM) unless otherwise indicated and *N* is the number of individual characters measured. Whenever possible, in all specimens, measurements of each character were averaged from measurements of characters taken from 5 different segments anteriad of the last mature segment. Measurements of characters in mature segments were taken from the last mature segment, defined as the segment immediately anterior to the observed segment in which eggs begin to appear in the developing uterus. Mean and standard deviation are given in parentheses. For measurements of egg characteristics, *N* represents the number of individual characters measured in the eggs (see Table 5).

**Table 5.** Measurements for Raillietina dowleri n. sp. found in Nesoryzomys swarthi on the island of Santiago, Galápagos, Ecuador. Measurements are in micrometers

	Strobila	Strobila	No. segments	Scolex W	Scolex L
	Max L	Max W			
	*N* = 7	*N* = 7	*N* = 7	*N* = 7	*N* = 7
Max	133	1569	489	384	473
Min	49	1137	250	289	244
Avg	95	1337	377	344	327
SD	32	139	93	36	80
CV	0·3	0·1	0·2	0·1	0·2
VAR	1022	19 372	8562	1315	6335
SE Mean	14·3	52·6	34·9	13·7	30·1
	Neck W	Neck L	Rostellum W	Rostellum L	No. of rostellar
					Hooks
	*N* = 7	*N* = 7	*N* = 7	*N* = 3	*N* = 7
Max	251	1580	141	94	153
Min	212	743	64	76	122
Avg	250	1122	93	84	138
SD	36	281	31	9	22
CV	0·1	0·2	0·3	0·1	0·1
VAR	1262	79 005	944	82	481
SE Mean	13·4	106·2	11·6	5·2	15·5
	Sucker	Sucker	Cirrus	Cirrus	No. Testes
	Max L	Max W	Sac L	Sac W	
	*N* = 8	*N* = 8	*N* = 7	*N* = 7	*N* = 3
Max	117	95	179	46	29
Min	91	64	106	33	22
Avg	108	88	137	42	25
SD	12	12	27	4·8	4
CV	0·1	0·1	0·2	0·1	0·1
VAR	145	142	721	22·6	14·3
SE Mean	4·3	4·2	10·2	1·8	2·2
	Testes L	Testes W	Ovary L	Ovary W	Vitelline
					Gland L
	*N* = 7	*N* = 7	*N* = 7	*N* = 7	*N* = 7
Max	46	37	107	201	74
Min	33	29	258	73	45
Avg	38	33	168	121	60
SD	6	3	60	48	10
CV	0·1	0·1	0·3	0·4	0·2
VAR	31	7·81	3556	2258	100
SE Mean	2·1	1·1	22·5	17·9	3·8
	Vitelline	Egg L	Egg W	Seminal	Seminal
	Gland W			Receptacle L	Receptacle W
	*N* = 7	*N* = 4	*N* = 4	*N* = 2	*N* = 2
Max	61	26	22	148	23
Min	37	22	18	117	18
Avg	45	24	20	133	21
SD	8	2	2	22	4
CV	0·2	0·1	0·1	0·1	0·1
VAR	64	3	3	481	13
SE Mean	3·01	0·82	0·85	15·5	2·5

## Description

### *Raillietina dowleri* n. sp

**Figure 1.**
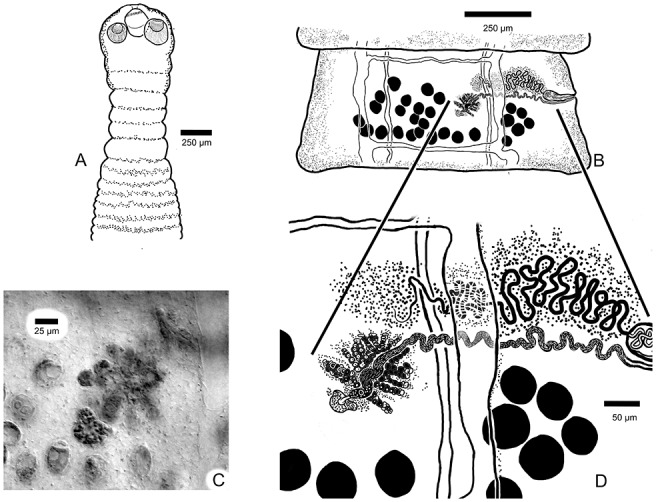
(A) Anterior end (Scolex and neck) of *Raillietina dowleri* n. sp., (B) Mature segment of *Raillietina dowleri* n. sp., ventral view. (C) Photographic image of testes to the left, vitelline gland center compact gland, and ovary with oöcapt and ovarian lobes evident. To the right of image c can be seen the ventral osmoregulatory duct running from top to bottom of image. (D) Expanded view of drawing of vitelline gland, ovary, vagina distal and of the cirrus sac, testes (black), and convoluted seminal duct (vas deferens). All ducts can be seen to pass between the dorsal and ventral osmoregulatory canals.

**Figure 2.**
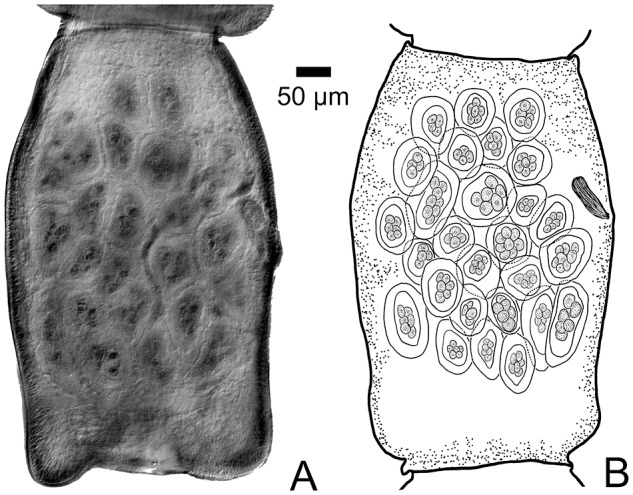
(A) Photograph of a gravid proglottid of *Raillietina dowleri* n. sp. showing the distribution of egg capsules and eggs. (B) Drawing of a gravid proglottid of *Raillietina dowleri* n. sp. showing the distribution of the egg capsules and the disposition of the cirrus sac being pushed anteriad by the developing egg capsules.

**Figure 3.**
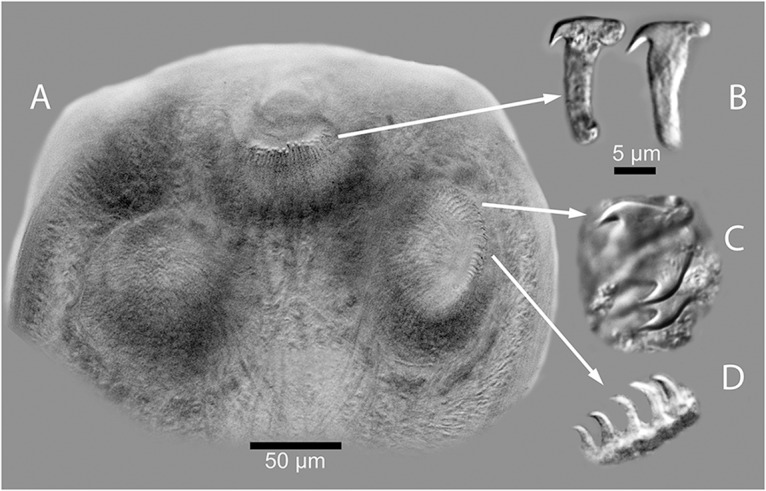
(A) Photograph of the anterior part of the Scolex of *Raillietina dowleri* n. sp., showing the hooks around the rostellum. (B) Expanded image of 2 of the hooks dissected out of the rostellum of the specimen shown in FIGURE 3A. (C) Image of the hooks lining the anterior part of the suckers, and (D) The small claw shaped hooks lining the posterior parts of the suckers of *Raillietina dowleri* n. sp.

For the following description, 7 full tapeworm specimens were studied. Scolex (Figures 1A; 3A–3C), *N* = 7, 289–384 (344 ± 36) in maximum width. Suckers, *N* = 8, 91117 (108 ± 12) long by 64–95 (88 ± 12) wide. Dorsal and ventral osmoregulatory canals join within Scolex at base of rostellum. Rostellum present and armed with approximately 140 claw hammer-shaped hooks, *N* = 8, 14–16 (15 ± 0.8) long (Figure 3B). Suckers armed with 2 types of hooks or spines showing both thicker falcate shaped hooks with recurved spines (Figure 3C) and thin, claw-shaped hooks (Figure 3D). Neck (Figure 1A), *N* = 7, 743–1580 (1122 ± 281) long by 212–251 (250 ± 36) in maximum width. Strobila, *N* = 7, 49–133 mM (94.6 ± 31.9 mM) long, with 250–489 (377 ± 93) segments; maximum width 1137–1569 (1337 ± 139) attained late in gravid segments (Figures 1B, 1C). Strobilae craspedote with intersegmental boundaries well-defined in both mature and gravid segments. Mature segments (Figure 1B) wider than long, gravid segments with developed egg capsules longer than wide (Figures 2A, 2B); strobila attenuated anteriad, with increase in relative length beginning in mature segments; length/width ratio of mature and gravid segments 0.20–0.34 (*N* = 7) and 0.29–1.69 (*N* = 7), respectively. Cirrus sac elongate, fusiform, *N* = 7, 106–179 (137 ± 27) in maximum length by 33–46 (42 ± 5) in maximum width. Cirrus unarmed. Testes, mostly round in overall shape, *N* = 7, 29–38 (38 ± 6) long by 29–37 (33 ± 3) wide, situated with most testes occurring in segment antiporal and only a few poral relative to the ovary (Figures 1B, 1C). Number of testes per mature proglottid *N* = 3, 22–29 (25 ± 4). Seminal receptacle, *N* = 2, 117–148 (133 ± 22) long by 18–23 (21 ± 4) in maximum width, extending porad, mostly anterior to ovary. Ovary (lobate, with small or large lobes), *N* = 7, 107–258 (168 ± 60) in maximum width by 73–201 (121 ± 48) in maximum length. Vitelline gland with relatively smooth margins, *N* = 7, 37–61 (45 ± 8) wide by 45–74 (60 ± 10) in maximum length, situated dorsal and posterior to ovary. Genital ducts always passing between excretory canals (Figures 1B, 1C). Eggs subspherical with thin outer shell, *N* = 4, 22–26 (24 ± 2) long by 18–22 (20 ± 2) wide. Egg capsules (Figures 2A, 2B) *N* = 2, 21–25 (22 ± 2), 4–8 eggs per capsule.

## Taxonomic summary

*Symbiotype host* (see Frey et al., 1992): Santiago Galápagos Mouse, *Nesoryzomys swarthi* Orr, 1938 (Rodentia: Cricetidae). Symbiotype Number: NMNH:USNM570194).

*Type Locality*: La Bomba, Santiago: Galápagos, Ecuador, 0°11.21–s; 90°42.04–W.

*Collection date*: 7 July 1999.

*Site of infection*: Small intestine, duodenum.

*Prevalence*: Five of twelve specimens of *Nesoryzomys swarthi* examined (33%).

*Specimens deposited*: Holotype: HWML217626, Field Collection Number: ASK5508; Paratypes: HWML217627, HWML217628, HWML217629, HWML217630, HWML217631, HWML217632, HWML217633, HWML217648; Additional specimens examined: HWML217634, HWML217635, HWML217636.

*Etymology*: This species was named after Robert C. Dowler, Professor of Biology, Emeritus, Angelo State University, San Angelo, Texas in honour of his long-term commitment to research in mammalogy, mammalian biodiversity, museum collections and mammalian parasitology. Without his dedication to this project and his leadership in collecting under rigorous field conditions, the occurrence and diversity of these species of parasites in the Galápagos would still remain unknown.

## Comparisons

The cestode genus *Raillietina* (Order Cyclophyllidea: Family Davaineidae) contains more than 200 described species with a cosmopolitan distribution in birds and mammals (Schmidt, 1986). However, because it is unlikely that any species of oryzomyine rodents have made it across the Pacific Ocean to the Indomalayan or Australasian zoogeographic regions and there is no evidence of this occurring, we restrict our comparisons to those species of *Raillietina* occurring in mammals of the Neotropical and southern Nearctic regions (see Table 6). In addition (as noted earlier), invasive rodents of the genera *Rattus* and *Mus* were collected from either the same localities or near the same areas as from where individuals of the endemic species of rodents were collected and no evidence of this new cestode species was discovered in any of the invasive murids.

**Table 6.** List of species of Raillietina from rodents and primates in the Caribbean, central, and South America. Measurements of characters from original papers are given

	*R. dowleri* n. sp.	*R. demerariensis*	*R. alouattae*	*R. trinitatae*	*R. guaricana*	*R. celebensis*	*R. oligocapsulata*	*R. multitesticulata* Perkins, 1950	*R. halli* Vigueras, 1943
				Cameron & Reesel, 1951	César and Luz, 1993				
			Baylis, 1947			(Baer and Sandars, 1956)	Sato et al., 1999		
		Daniels, 1895 From Sato et al., 1999							
**Character**									
**name**									
Total length	49–133 mM	320–660 mM	130–340 mM	60 mM	300–601 mM	676–898 mM	28–160 mM	270–330 mM	90–120 mM
Max. width of strobila	1137–1569	2800–3000	—	—	—	2200	240–500	—	1,300–1,800
Scolex length	244–473	140–160	380–470	320	250–300	—	168–212	—	420
Scolex width	289–384	276–308	450–620	370	240–380	—	280–296	390–570	480
Number of hooks	124–140	162–184	170–224	170	66–78	120–126	170	—	200–220
Hook length	14–16	15–19	—	—	10–20	18–23	—	—	18–20
Neck width	212–251	224–360	—	—	—	—	208–240	290–350	1,200–1,600
Suckers L × W	64–117	52–132	—	88–100	80–110	—	96–120	120–153	120–130
No. of proglottids	250–489	582–806	—	—	947	—	461–500	—	-
No. of testes/proglottid	22–29	44–73	110–130	28–32	26–41	20–37	55–73	115–120	50–70
Testes size	29–38	40–90	60–80	35	20–70	17–50	26–44	60–80	45–60
Ovary length	107–258	320–450	—	—	—	60–150	200–240	—	-
Ovary width	73–201	450–500	300	200	50–300	90–180	120–192	345–4	-
Vitelline gland W	45–60	120–160	150	90	60–180	40–100	56–112	-	-
Egg L × W	18–26	22–36	14–20	10	15	20–40	20–40	50–60	14–16
No. egg capsules/proglottid	21–25	234–331	38–55	50–70	92–121	135–180	22–24	47–80	40–60
Eggs/Capsule	4–8	20	6–11	8–12	4–12	2–8	15–20	3–8	8–15
Type host	*Nesoryzomys* s*warthi*	*Agouti paca*	*Alouatta*	*Cuniculus paca*	*Sooretamys angouya*	*Rattus norvegicus*	*Sylvilagus* *brasiliensis*	*Alouatta seniculus*	*Capromys pilorides*
**Geographic** **locality**	Isla Santiago Galápagos	Ecuador, Guiana, Cuba, Venezuela	Paramaribo, Suriname	Trinidad	Guaricana, Parana, Brazil	Sao Gancala RJ Brazil	Coyoweteri, Sierra Parima, T.F.A., Venezuela	Kongarooma, Guiana	Cuba

## Differential diagnosis

*Raillietina dowleri* n. sp. can be recognized as distinct from *R. demerariensis* Daniels, 1895, described from the red howler monkey, *Alouatta seniculus* (Linnaeus, 1766) in South America based on the width of the strobila; *R. dowleri* n. sp. has a much larger strobilar width with a mean width of 1, 337 µM, whereas the maximum width of the strobila of *R. demeriensis* does not exceed 640 µM (Stunkard, 1953). In addition, *R. dowleri* n. sp. can be recognized as distinct from *R. alouattae* Baylis, 1947, described from the Guyanan red howler monkey *Alouatta macconelli* (Linnaeus, 1766) also from South America, by possessing many fewer testes: *R. dowleri* n. sp. has from 22–29 testes in each mature proglottid whereas *R. alouattae* sports 110–130 in each mature proglottid.

*Raillietina dowleri* n. sp. can be recognized as distinct from *R. trinitatae* Cameron and Reesel, 1951, described from the Paca, *Cuniculus paca* (Linnaeus, 1766), from the island of Trinidad in the Caribbean, in having much larger eggs: Eggs of *R. dowleri* n. sp., are 22–26 µM by 18–22 µM while gravid proglottids of *R. trinitatae* have eggs that average only about 10 µM in width. In addition, *R. dowleri* possesses from 4 to 8 eggs per egg capsule and only 21–25 egg capsules per gravid proglottid compared to 50–70 egg capsules with 8–12 eggs per capsule in *R. trinitatae*. The rostellar hooks of *R. dowleri* are claw-hammer shaped (Figures 3B-3D) while those of *R. trinitatae* are a single fork shape [see Fig. 6 in Cameron and Reesel (1951)].

*Raillietina dowleri* n. sp. can be recognized as distinct from *R. guaricana* (César and Luz, 1993) described from *Sooretamys angouya* (Fisher, 1814) [syn. *Oryzomys ratticeps* (Hensel, 1872)] in Brazil, in having many fewer hooks on the rostellum with from 120 to 140 rostellar hooks occurring in *R. dowleri* vs only 66–78 in *R. guaricana*; in having a much smaller strobila, both in length and maximum width, smaller size of suckers, and by the much smaller size of the egg capsules which range from 21 to 25 µM in *R. dowleri* compared to 92–121 µM in *R. guaricana* (see César and Luz, 1993).

From *R. halli* (Vigueras, 1943) collected from *Capromys pilorides* (Say, 1822) in Cuba in the early 1940’s, *R. dowleri* n. sp. can be recognized as distinct by having fewer hooks on the rostellum, with *R. halli* possessing from 200 to 220 hooks, while *R. dowleri* has only from 120 to 140 hooks on the rostellum, while each gravid proglottid of *R. halli* contains from 40 to 60 egg capsules, compared to 21–25 per proglottid as found in *R. dowleri* [see Vigueras (1943) for a complete description of this species].

*Raillietina dowleri* n. sp. can be recognized as distinct from *R. celebensis* (Baer and Sandars, 1956) originally described from *Rattus norvegicus* by having a much shorter strobila, shorter hooks on the rostellum, and many fewer egg capsules per gravid proglottid. For additional information on *R. celebensis*, see Baer and Sandars (1956), and the re-description by de Oliveira et al. (2017).

*Raillietina dowleri* n. sp. can be recognized as distinct from *R. oligocapsulata* (Sato et al., 1999) described from the tapeti or forest cottontail rabbit [cf. *Sylvilagus brasiliensis* (Linnaeus, 1758)] based on the number of hooks on the rostellum (124–140 in *R. dowleri* vs 170, in *R. oligocapsulata*) number of eggs per egg capsule, having 4–8 eggs/capsule whereas *R. oligocapsulata* has 15–20 eggs/capsule (see description by Sato et al., 1998).

Finally, *R. dowleri* n. sp. can be recognized as distinct from *R. multitesticulata* Perkins, 1950, described from the Colombian red howler monkey (*Alouatta seniculus*) collected near Kongarooma in the former British Guiana based on number of testes with *R. dowleri* sporting from 22 to 29 testes in each mature proglottid whereas *R. multitesticulata* has 115–120 testes in each proglottid (Perkins, 1950).

## Summary of additional species of parasites recovered from rodents collected

Phylum Nemata

Physalopteridae

*Physaloptera calnuensis* (Sutton, 1989)

*Locality, deposition and host records*: Santa Fe: Galápagos, Ecuador, 0°48.21ʹS 90°2.45ʹW, 16 July 1999, 2 males (HWML17007) from *Aegialomys galapagoensis;* Volcan Wolf, Isabela: Galápagos, Ecuador, 0°3.96ʹN 91°24.18ʹW, 7 September 1999, 2 males and 3 females (HWML17053) from *Rattus rattus*.

*Remarks*: Sutton’s type host for *P. calnuensis* was *Calomys laucha* (Fischer, 1814) from the stomach (Sutton, 1989). *Physaloptera calnuensis*, originally described from *Calomys laucha* from Argentina may have transferred to the Galápagos with the original endemic rodents. The existence of this nematode in *Rattus* in the islands may indicate ecological fitting from endemic rodents to the muroid invaders.

*Prevalence: Physaloptera calnuensis* occurs in 1 of 12 specimens of *A. galapagoensis* examined (8·83%) and from 1 of 22 specimens of *R· rattus* examined (4·55%).

Spiruridae

*Mastophorus muris* (Gmelin, 1790)

*Locality, deposition, and host records*: Cabo Douglas, Fernandina: Galápagos, Ecuador, 1°18.24ʹS 91°39.14ʹW, 7 November 1999, 6 females/4 juveniles (HWML17049, HWML17052, HWML17050, HWML17047, HWML17046, HWML17048) from *Nesoryzomys fernandinae*; Santa Fe: Galápagos, Ecuador, 0°48.21ʹS 90°2.45ʹW, 16 July 1999, 2 females (HWML17013) from *Aegialomys galapagoensis*; East of Eden, Santa Cruz: Galápagos, Ecuador, 0°33ʹ40.2114ʹʹ–90°31ʹ40.8ʹʹ, 15 July 1999, 3 females (HWML17016, HWML17017) from *Rattus rattus*; South of Cerro Bruho, San Cristobal: Galápagos, Ecuador, 0°47ʹ6ʹʹ–89°28ʹ5.8794ʹʹ, 24 July, 1999, 6 females/7 males (HWML17012) from *Rattus rattus*; West of Punta Pitt, San Cristobal: Galápagos, Ecuador, 0°42ʹ43.1994ʹʹ–89°15ʹ11.8794ʹʹ, 25 July 1999, 3 specimens (HWML17014) from *Rattus rattus*.

*Remarks*: Gmelin’s type host for *M. muris* was *Myodes glareolus* Gmelin 1780, (see: Quentin, 1971). It appears that this species of nematode now occurs in endemic mammals after host-switching from invasive *Rattus* or *Mus*.

*Prevalence*: We found these nematodes in 6 of 12 *N. fernandinae* examined (50%); 1 of 12 *A. galapagoensis* examined (8·33%); 4 of 22 *R. rattus* examined (18·18%).

*Protospirura numidica* Seurat, 1914

*Locality, deposition and host records*: North of Cerro Bruho, San Cristobal: Galápagos, Ecuador, 0°44ʹ44.988ʹʹ–89°26ʹ22.92ʹʹ, 26 July 1999, 3 females (HWML118823) from *Rattus rattus*.

*Remarks*: Seurat’s type host for *P. numidica* was *Felis ocreata* Bate, 1905, from the stomach of the cat (Crook and Grundmann, 1964).

*Prvalence*: 1 of 22 *R. rattus* examined (4·55%).

Phylum Platyhelminthes

Hymenolepididae

*Hymenolepis diminuta* (Rudolphi, 1819)

*Locality, deposition and host records*: Volcan Wolf, Isabela: Galápagos, Ecuador, 0°3.96ʹN 91°24.18ʹW, September 7, 1999, 1 specimen (HWML217637) from *Rattus rattus*; La Bomba, Santiago: Galápagos, Ecuador, 0°11ʹ12.5874ʹʹ–90°42ʹ2.5194ʹʹ, 7 July 1999, 4 individuals (HWML217638, HWML217639, HWML217640) from *Rattus rattus*; North of Cerro Bruho, San Cristobal: Galápagos, Ecuador, 0°44ʹ44.988ʹʹ–89°26ʹ22.92ʹʹ, 26 July 1999, 1 individual (HWML217641) from *Rattus rattus*.

*Remarks*: Rudolphi’s listed type hosts for *H. diminuta* include *Rattus rattus* and *Mus musculus* and this cestode is a common parasite of the small intestine of rodents (Oldham, 1931; Gardner and Schmidt 1986; Dursahinhan et al., 2023). In addition to the discovery of these cestodes in invasive rats in the Galápagos, it is interesting to note that this cestode is found in rodents (especially species of the genus *Rattus*) world-wide, probably having been distributed globally by humans with their synanthropic species of *Rattus*. Thus, the presence of these cestodes in *Rattus* on Santiago Island can probably be attributed to natural infections in the invasive rats; however, it is interesting to note that no instances of *H. diminuta* are known from the endemic species of rodents that were sampled.

*Prvalence*: 5 of 22 *R. rattus* examined (22·73%).

Taeniidae

*Taenia taeniaeformis* (Batsch, 1786)

*Locality, deposition and host records:* Volcan Wolf, Isabela: Galápagos, Ecuador, 0°3.96ʹN 91°24.18ʹW, 7 September 1999, 2 individuals (HWML217642, HWML217643) from *Rattus rattus*; Caleta Iguana, Cerro Azul, Isabela: Galápagos, Ecuador, 0°55ʹ42.0954ʹʹ–91°23ʹ36.96ʹʹ, 13 July 1999, 4 individuals (HWML217647) from *Rattus rattus*; South of Cerro Bruho, San Cristobal: Galápagos, Ecuador, 0°42ʹ43.1994ʹʹ–89°15ʹ11.8794ʹʹ, 24 July 1999, 1 individual (HWML217646) from *Rattus rattus*; West of Punta Pitt, San Cristobal: Galápagos, Ecuador, 0°42ʹ43.1994ʹʹ–89°15ʹ11.8794ʹʹ, 25 July 1999, 1 individual (HWML217644) from *Rattus rattus*; North of Cerro Bruho, San Cristobal: Galápagos, Ecuador, 0°44ʹ44.988ʹʹ–89°26ʹ22.92ʹʹ, 26 July 1999, 1 individual (HWML217645) from *Rattus rattus*.

*Remarks*: Batsch’s type host for *T. taeniaeformis* was *Felis* sp. This cestode has a worldwide distribution with adults in cats and rodents serving as intermediate hosts.

*Prevalence*: 8 of 22 *R. rattus* examined (36·36%). These findings indicate that feral cats on the islands are consuming *R. rattus* and these rodents are living in a commensal relationship with cats.

## Discussion

Both classical and evolutionary parasitology has been understudied in the Galápagos even though it is such an important geographic location for the development of the theory of evolution. The present paper starts to alleviate this dearth of information on parasites, at least in mammals, by outlining occurrence and prevalence of endoparasites in both endemic and invasive species of rodents. However, since few specimens were collected, examined and necropsied, and only metazoan parasites were preserved (see also Gettinger et al., 2011) the true parasite diversity within the Galápagos rodent fauna is still not well-known and remains understudied.

Additional collecting and analysis of parasites from both introduced and endemic mammals and birds would shed light on their transmission dynamics as shown by levels of network connections and would enable a local and robust network analysis (e.g. Dursahinhan et al., 2023) using both occurrence data at the species level as well as levels of connectedness that would be shown in a molecular phylogeographic analysis.

However, unless well-trained (in field methods) mammalogists/parasitologists are involved with field collections, endoparasites (helminths and protozoa) as well as ectoparasites are almost never actually collected nor are they considered as important components of the ecological communities of rodents or other mammals. Or they are collected as an afterthought, with little effort being made to preserve specimens of parasite of high quality that can be used for both morphology and molecular investigations into the future.

The intrinsic value of parasites cycling in natural ecosystems is a difficult parameter to estimate, mostly because the majority of biologists think of parasites as unattractive, unappealing and unnecessary inhabitants of their favourite animal groups or species. In fact, the first thing that many field biologists do when they begin to prepare a specimen for a museum study skin is to discard the intestinal tracts of any specimens collected (Gardner, pers. obs.). This occurs now on a regular basis despite the continued and relatively recent calls for training and the fact that there are available published papers that outline methods and provide examples of the importance of collecting parasites from their associated vertebrate hosts [see: (Gardner, 1996) (mammals); (Gardner and Jiménez-Ruiz, 2009) (bats); (Gardner et al., 2012) (herps); (Galbreath et al., 2019) (mammals)]. This appalling destruction of a significant portion of the biodiversity of potentially endangered or rare species in an area that is being surveyed for preservation or conservation purposes is significant as parasites have been shown to have not only intrinsic value to natural ecosystems, but extrinsically, these organisms can serve as indicators of ecological health (Marcogliese, 2005) as well as probes for current as well as ancient biodiversity (Gardner and Campbell, 1992).

Following the Document, Assess, Monitor, Act protocol (Brooks et al., 2014) we call for more parasite surveys on the mammalian fauna of the Neotropics followed by subsequent phylogenetic studies to be completed on these cestodes (*Raillietina* spp.) on mainland South America and the Galápagos Islands before the habitats are forever obliterated by the continued encroachment by humans and their machines into residual natural areas. A phylogenetic/phylogeographic analysis including all known species of *Raillietina* using both morphology and molecules would give deeper insight into whether *Raillietina dowleri* from *Nesoryzomys swarthi* is derived from a direct ancestral invasion of the islands of its rodent hosts or the presence of these cestodes in individuals of *N. swarthi* is the result of ecological fitting in the archipelago that occurred after the establishment of rice-rats in the islands. Parent et al. (2008) point out that most of the terrestrial fauna diversified in parallel to the geological formation of the islands, so it is to be expected that there is more diversity of these tapeworms and their associated hosts than has yet been recorded. At this point, with no information on the helminth-parasite fauna of the passerines of the Galápagos and only limited collections that were made of the rodents, the level of parasite biodiversity of the rodent fauna of the islands is still relatively unknown.

## Data Availability

All specimens of helminths collected and analysed herein are freely available for study at the Harold W. Manter Laboratory of Parasitology: Contact information is provided at the permanent web site of the Manter Lab. See: https://hwml.unl.edu.
